# Eravacycline pharmacokinetics/pharmacodynamics in the hollow fiber system model of *Mycobacterium abscessus* lung disease

**DOI:** 10.1128/spectrum.03432-25

**Published:** 2025-12-10

**Authors:** Sanjay Singh, Avneesh Shrivastava, Gunavanthi D. Boorgula, Mary C. Long, Brian Robbins, Tawanda Gumbo, Shashikant Srivastava

**Affiliations:** 1 Division of Infectious Diseases, Department of Medicine, University of Texas at Tyler School of Medicine, Tyler, Texas, USA; 2 Advanta Genetics, Tyler, Texas, USA; 3 IMPI Biotech Consortium Inc., Tateguru, Zimbabwe; 4 Department of Cellular and Molecular Biology, The University of Texas Health Science Centre at Tyler, Tyler, Texas, USA; Innovations Therapeutiques et Resistances, Toulouse, France

**Keywords:** MIC, probability of target attainment, Monte Carlo experiments

## Abstract

Treatment of *Mycobacterium abscessus* (MAB) pulmonary diseases is challenging due to intrinsic resistance to many antibiotics and requires prolonged intravenous therapy with drugs that have severe side effects. The treatment guidelines include tetracyclines as part of the combination. We tested a 3rd generation tetracycline, eravacycline, for its potential role in MAB pulmonary disease. Eravacycline minimum inhibitory concentration (MIC) (ATCC#19977 strain and 59 clinical isolates) ranged between 0.0075 and 0.3 mg/L, and the MIC_50_ and MIC_90_ were 0.15 and 0.3 mg/L, respectively. In the static concentration-response studies, eravacycline killed 5.58 + 0.34 log_10_ CFU/mL MAB, and EC_50_ (concentration associated with 50% of the maximal effect [*E*_max_]) was 0.05 ± 0.01 mg/L. In the hollow fiber system model of *Mycobacterium abscessus* (HFS-MAB), examining eight eravacycline human-equivalent lung exposures, the ratio of area under the concentration-time curve to MIC (AUC_0-24_/MIC) of 3,588 was identified as EC_80_ or the optimal exposure target for MAB kill. The probability of target attainment even with eravacycline doses of 4 mg/kg twice a day (8 mg/kg/day) falls below 90% at an MIC of 0.125 mg/L, and the cumulative fraction of response was only 30.56% in the 10,000 virtual patient trial. In summary, a MIC pattern does not accurately inform whether a tetracycline (in this study, eravacycline) has good efficacy, which must be determined using preclinical models applying a pharmacokinetics/pharmacodynamics study design. The HFS-MAB results suggest that, despite low MICs, the probability of better therapeutic outcomes with eravacycline is limited.

**IMPORTANCE:**

Treatment of *Mycobacterium abscessus* (MAB) pulmonary diseases is challenging due to intrinsic resistance to many antibiotics. The ATS/IDSA guidelines include tetracyclines as part of the combination. Eravacycline is a new tetracycline approved for the treatment of complicated intra-abdominal bacterial infections with better tolerability and low gastrointestinal adverse events compared to tigecycline. We performed an eravacycline pharmacokinetics (PK)-pharmacodynamics (PD) study for dose optimization with respect to the MAB pulmonary disease mimicking the intrapulmonary PKs in the hollow fiber model system of MAB (HFS-MAB). While eravacycline MICs and static concentrations suggest great potential and even activity better than omadacycline and tigecycline, the drug only killed 0.56 log_10_ CFU/mL below stasis in the HFS-MAB. Monte Carlo simulation experiments suggested that optimal doses for MAB pulmonary disease are likely in the range that would not be tolerated by patients.

## INTRODUCTION

*Mycobacterium abscessus* (MAB) is a fast-growing nontuberculous mycobacteria (NTM) associated with chronic pulmonary disease (1). There are three subspecies reported commonly as causing disease, MAB subsp. *abscessus*, MAB subsp. *massiliense*, and MAB subsp. *bolletii* (1). Treatment of MAB pulmonary diseases is challenging due to intrinsic resistance to many antibiotics, requiring prolonged intravenous (IV) therapy with drugs that have severe side effects (2, 3). As a result, the treatment regimens for MAB pulmonary disease vary widely (4). The ATS/IDSA guidelines include tetracyclines as part of the combination. Tetracyclines inhibit bacterial protein synthesis and have been shown to have clinical benefits in patients with MAB pulmonary diseases (5, 6). Eravacycline is a new tetracycline approved for the treatment of complicated intra-abdominal bacterial infections in patients >18 years of age (7). Emerging reports suggest that MAB is susceptible to eravacycline (8–10). Early reports suggest that eravacycline has better tolerability and low (14%) gastrointestinal adverse events compared with tigecycline (24%) (11, 12). However, eravacycline pharmacokinetics (PK)-pharmacodynamics (PD) study for dose optimization with respect to the MAB lung disease is lacking.

Here, we employed the hollow fiber system model of MAB (HFS-MAB), a preclinical drug development tool approved by the European Medicines Agency (EMA) and editorially endorsed by the U.S. Food and Drug Administration (13–16), to perform eravacycline PK/PD studies. The HFS-MAB, an adaptation of the model we have extensively used for TB drug development (17), is a PK system with the ability to mimic human-like concentration-time profiles of a single drug or multidrug combination with different half-lives. Briefly, in its simplest form of the continuous dilution system, bacteria grow in the peripheral compartment of a hollow fiber cartridge housing semipermeable hollow fiber membranes for which the material can vary by drug properties and pores allow exchange of nutrients and drugs in and out of the peripheral compartment while preventing bacteria from leaving the peripheral compartment. Drugs are infused into the central compartment and circulate through the system using peristaltic duet pumps. The fresh media inflow and used media outflow rates allow the simulation of drug half-life. The peripheral compartment is sampled over time to measure the changes in the bacterial burden in response to the drug treatment and emergence of drug resistance, if any. Samples from the central compartment are used to validate the drug concentration-time profile to determine the actual drug exposure achieved in the system.

The currently recommended dose of eravacycline to treat complicated intra-abdominal bacterial infection is 1 mg/kg administered every 12 h (Q12h), administered as an intravenous infusion over 60 min twice daily for up to 14 days, with low drug-related adverse events (11). The peak (*C*_max_) plasma concentration following 1 mg/kg single intravenous infusion has been reported as 2.13 mg/L and the area under the concentration-time curve (AUC_0-12_) as 4.31 mg*h*L^−1^ or a free-drug (*f*) AUC_0-12_ of 0.77 mg*h*L^−1^ (7). Eravacycline protein binding (79% to 90%) increases with an increase in concentration (7). The eravacycline population PKs are best described by a three-compartment model, with a central compartment (systemic circulation), a peripheral compartment, and a lung compartment (18). The eravacycline epithelial lining fluid (ELF)-to-plasma concentration is proportional to 8.26 times free-drug concentration (18). Thus, the 1 mg/kg will have an ELF AUC_0-24_ of 13.24 mg*h*L^−1^. Here, we used these intrapulmonary PKs to perform an eravacycline PK/PD study in the HFS-MAB.

## RESULTS

### Static concentrations versus effect

The eravacycline MIC against the reference American Type Culture Collection 19977 (ATCC#19977) isolate was 0.15 mg/L. We performed static concentration-response studies on this isolate and cultured the samples on Middlebrook 7H10 agar for CFU estimation after 72 h of incubation in cation-adjusted Mueller Hinton broth (CAMHB) at 30°C under shaking conditions. The inhibitory sigmoid maximal effect (*E*_max_) model fit parameter estimate for bacterial burden in the nontreated control (*E*_con_) was 5.31 ± 0.25 log_10_ CFU/mL, *E*_max_ compared with *E*_con_ was 5.58 ± 0.34 log_10_ CFU/mL, *H* (Hill coefficient) was 0.87 ± 0.12, and EC_50_ (concentration associated with 50% of the *E*_max_) was 0.05 ± 0.01 mg/L, which is 0.33× of the MIC (*r*^2^ = 0.97).

### HFS-MAB exposure-effect studies

**Fig 1:**
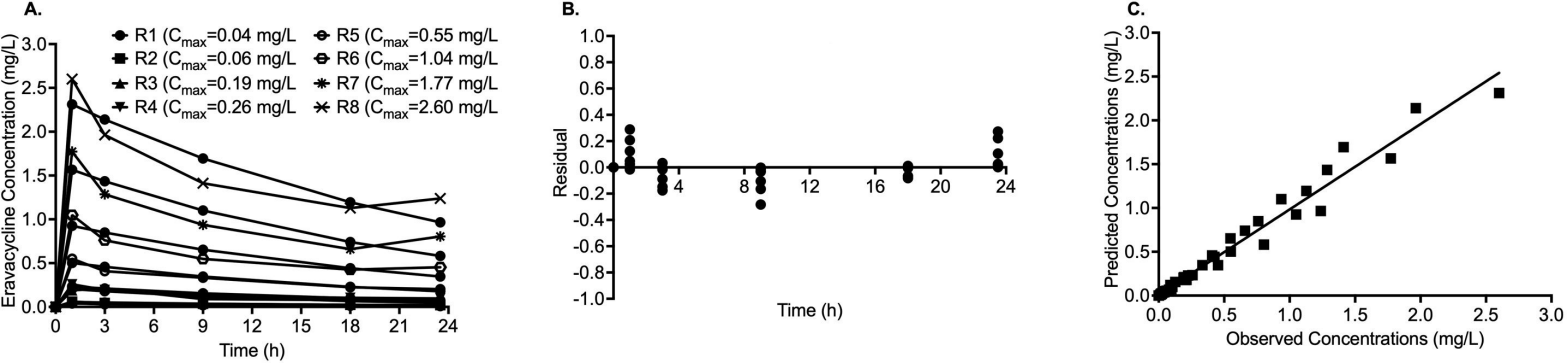
Eravacycline pharmacokinetics/pharmacodynamics in the HFS-MAB. (**A**) Concentration-time profile of eravacycline with different doses administered once daily. (**B**) Residuals from the pharmacokinetic modeling of the HFS-MAB measured eravacycline concentrations showing a good model fit. (**C**) Linear regression between the HFS-MAB measured eravacycline concentrations and model-predicted concentrations.

The eravacycline concentration-time profiles with each of the eight doses in the HFS-MAB were as shown in Fig. 1A. The residuals from the PK model are shown in Fig. 1B, and regression between the measured and PK-modeled concentration in Fig. 1C shows a good model fit with minimal bias (slope 0.97; 95% confidence intervals [CIs]: 0.9249 to 1.024; *r*^2^ = 0.97). Mean ± standard deviation eravacycline clearance rate was 0.03 ± 0.001 L/h, volume of distribution was 0.58 ± 0.11 L, and half-life achieved in the HFS-MAB units was 14.20 ± 2.5 h. Table 1 summarizes the *C*_max_ and AUC_0-24_ with each eravacycline dose tested in the HFS-MAB and the ratio of *C*_max_/MIC and AUC_0-24_/MIC.

**TABLE 1:** Intrapulmonary eravacycline exposures equivalent achieved in the HFS-MAB

HFS-Mab ID	*C*_max_ (mg/L)	*C*_max_/MIC	AUC_0-24_	AUC_0-24_/MIC
R1	0.04	0.29	0.47	3.13
R2	0.06	0.40	0.77	5.14
R3	0.21	1.42	2.51	16.70
R4	0.26	1.72	3.13	20.88
R5	0.55	3.67	7.15	47.65
R6	1.05	7.00	13.05	87.00
R7	1.77	11.82	21.82	145.47
R8	2.60	17.33	33.93	226.20

**Fig 2:**
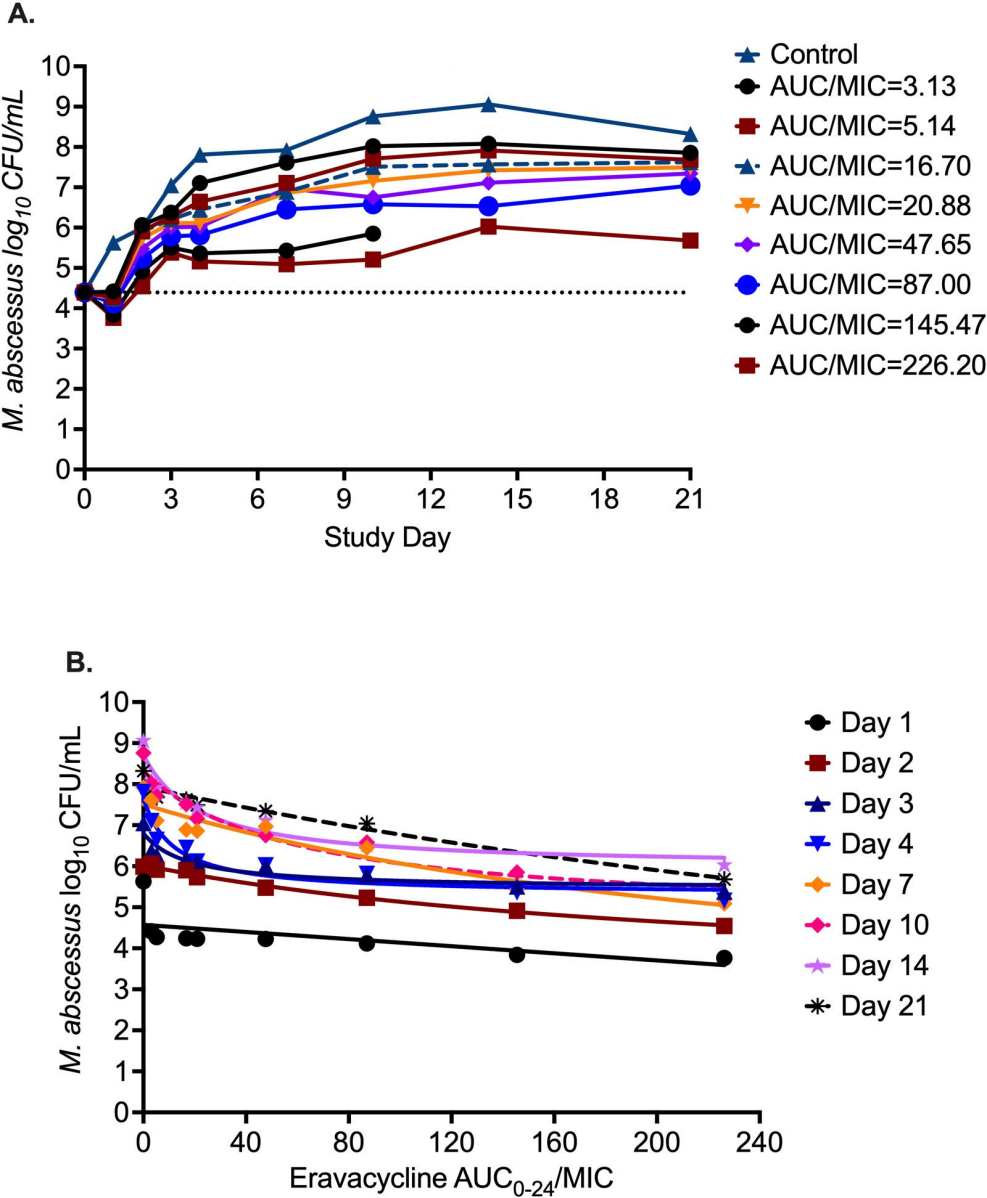
Eravacycline pharmacokinetics/pharmacodynamics in HFS-MAB. (**A**) Eravacycline time-kill curves with fluctuating drug concentrations using a once-daily dosing schedule. Eravacycline MIC of the reference ATCC#19977 strain was 0.15 mg/L. The HFS-MAB unit with AUC_0-24_/MIC of 145.47 showed contamination on study day 14; therefore, no data for this system were available for the later time points. (**B**) The inhibitory sigmoid maximal effect model fits for each sampling day to determine the relationship between eravacycline exposures and MAB bacterial burden.

The MAB kill curves with eight different eravacycline exposures in the HFS-MAB are shown in Fig. 2A. The HFS-MAB unit with AUC_0-24_/MIC of 145.47 showed fungal contamination on study day 14; hence, any data beyond this time point were removed from the analysis. The bacterial burden in the inoculum (*B_0_*) was 4.39 log_10_ CFU/mL, which grew to 8.32 log_10_ CFU/mL in 21 days. All eravacycline exposures failed to kill MAB below *B_0_*, except the two highest exposures (AUC_0-24_/MIC of 145.47 and 226.20), which killed 0.56 log_10_ CFU/mL below *B_0_* during the first 2 days of the study. However, since the study did not attempt to capture the emergence of drug resistance by culturing the samples on 3× MIC-supplemented agar, we are unsure about eravacycline resistance dynamics in HFS-MAB. On study day 10, the maximum effect compared to non-treated controls (*E*_con_) on the same day was recorded as 3.54 log_10_ CFU/mL. The inhibitory sigmoid maximal effect model results describing the relationship between eight eravacycline exposures and MAB burden at different sampling time points are shown in Fig. 2B. Table 2 summarizes the model parameters for each sampling day. The eravacycline median EC_80_ (exposure target for MAB kill) was calculated as an AUC_0-24_/MIC of 3,588.

**TABLE 2:** Summary of the inhibitory sigmoid maximal effect model parameters for each sampling timepoint to study the relationship between the eravacycline exposures (AUC_0-24_/MIC) and bacterial burden^*a*^

Parameter	Day 1	Day 2	Day 3	Day 4	Day 7	Day 10	Day 14	Day 21
Best-fit values								
*E*_con_	5.628	6.0	7.05	7.68	7.886	8.740	9.054	8.204
*E*_max_	1.789	1.66	6.76	3.18	6.001	6.764	4.494	6.576
*H*	0.60	1.43	0.25	0.55	0.5419	0.4689	0.4493	0.5769
EC_50_	1.275	86.27	31157	37.72	446.1	298.1	65.96	849.7
95% CIs								
*E*_con_	5.39 to 5.87	5.88 to 6.11	6.89 to 7.20	7.35 to 7.87	7.29 to 8.48	8.38 to 9.11	8.83 to 9.28	7.54 to 8.87
*E*_max_	1.47 to 2.10	1.19 to 2.12	−32.17 to 45.68	1.47 to 4.89	−11.84 to 23.84	−2.07 to 15.60	1.84 to 7.15	−22.69 to 35.84
*H*	0.26 to 0.96	0.69 to 2.18	−0.07 to 0.5610	0.17 to 0.92	−0.25 to 1.34	0.12 to 0.82	0.18 to 0.71	−0.4 to 1.56
EC_50_	0.42 to 2.97	44.19 to 128.3	−1,110,247 to 1,172,562	−45.13 to 120.6	−4,097 to 4,989	−1,306 to 1,902	−120.6 to 252.5	−10,538 to 12,237
Goodness of fit								
*r*^2^	0.959	0.98	0.98	0.98	0.92	0.98	0.99	0.95
AICc	−30.63	−35.77	−35.21	−23.60	−5.396	−16.31	−20.54	1.226

^*a*^ *E*_con_ and *E*_max_ in log_10_ CFU/mL, EC_50_ in mg/L, AICc is the corrected Akaike criteria score.

### MICs in 60 MAB isolates

The eravacycline MICs among the 60 isolates (1 ATCC plus 59 others) ranged between 0.0075 and 0.3 mg/L (Table 3). Among the 59 clinical isolates, 36 were MAB subsp*. abscessus*, 3 were MAB subsp*. bolletii,* and 21 were *MAB* subsp*. massilliense*. The MIC_50_ was 0.15 mg/L, and the MIC_90_ was 0.3 mg/L. This MIC distribution was used in dose-finding Monte Carlo experiments (MCEs).

**TABLE 3:** Eravacycline MIC distribution

Clinical isolates by subspecies	MIC (mg/L)
*M. abscessus* subsp. *abscessus*	0.15
*M. abscessus* subsp. *abscessus*	0.3
*M. abscessus* subsp. *abscessus*	0.3
*M. abscessus* subsp. *abscessus*	0.3
*M. abscessus* subsp. *abscessus*	0.15
*M. abscessus* subsp. *abscessus*	0.15
*M. abscessus* subsp. *abscessus*	0.15
*M. abscessus* subsp. *abscessus*	0.15
*M. abscessus* subsp. *abscessus*	0.3
*M. abscessus* subsp. *abscessus*	0.3
*M. abscessus* subsp. *abscessus*	0.015
*M. abscessus* subsp. *abscessus*	0.3
*M. abscessus* subsp. *abscessus*	0.3
*M. abscessus* subsp. *massilliense*	0.075
*M. abscessus* subsp. *massilliense*	0.15
*M. abscessus* subsp. *massilliense*	0.075
*M. abscessus* subsp. *massilliense*	0.3
*M. abscessus* subsp. *massilliense*	0.15
*M. abscessus* subsp. *massilliense*	0.075
*M. abscessus* subsp. *massilliense*	0.3
*M. abscessus* subsp. *bolletii*	0.3
*M. abscessus* subsp. *massilliense*	0.3
*M. abscessus* subsp. *abscessus*	0.0075
*M. abscessus* subsp. *abscessus*	0.0075
*M. abscessus* subsp.* massilliense*	0.075
*M. abscessus* subsp.* massilliense*	0.3
*M. abscessus* subsp.* abscessus*	0.15
*M. abscessus* subsp.* massilliense*	0.15
*M. abscessus* subsp.* abscessus*	0.0075
*M. abscessus* subsp.* abscessus*	0.15
*M. abscessus* subsp.* abscessus*	0.15
*M. abscessus* subsp.* abscessus*	0.075
*M. abscessus* subsp.* abscessus*	0.15
*M. abscessus* subsp.* abscessus*	0.3
*M. abscessus* subsp.* bolletii*	0.3
*M. abscessus* subsp.* bolletii*	0.3
*M. abscessus* subsp.* massilliense*	0.15
*M. abscessus* subsp.* massilliense*	0.3
*M. abscessus* subsp.* massilliense*	0.15
*M. abscessus* subsp.* massilliense*	0.3
*M. abscessus* subsp.* massilliense*	0.075
*M. abscessus* subsp.* massilliense*	0.075
*M. abscessus* subsp.* abscessus*	0.3
*M. abscessus* subsp.* massilliense*	0.0075
*M. abscessus* subsp.* abscessus*	0.075
*M. abscessus* subsp.* abscessus*	0.075
*M. abscessus* subsp.* abscessus*	0.15
*M. abscessus* subsp. *abscessus*	0.0075
*M. abscessus* subsp. *abscessus*	0.3
*M. abscessus* subsp. *abscessus*	0.3
*M. abscessus* subsp. *abscessus*	0.3
*M. abscessus* subsp. *massilliense*	0.3
*M. abscessus* subsp. *massilliense*	0.3
*M. abscessus* subsp. *abscessus*	0.075
*M. abscessus* subsp. *abscessus*	0.3
*M. abscessus* subsp. *abscessus*	0.075
*M. abscessus* subsp. *abscessus*	0.015
*M. abscessus* subsp. *abscessus*	0.037
*M. abscessus* subsp. *massilliense*	0.15
*M. abscessus* subsp. *abscessus*	0.15
MIC range	0.0075 to 0.3
MIC_50_	0.15
MIC_90_	0.3

### Dose-finding MCEs

**Fig 3:**
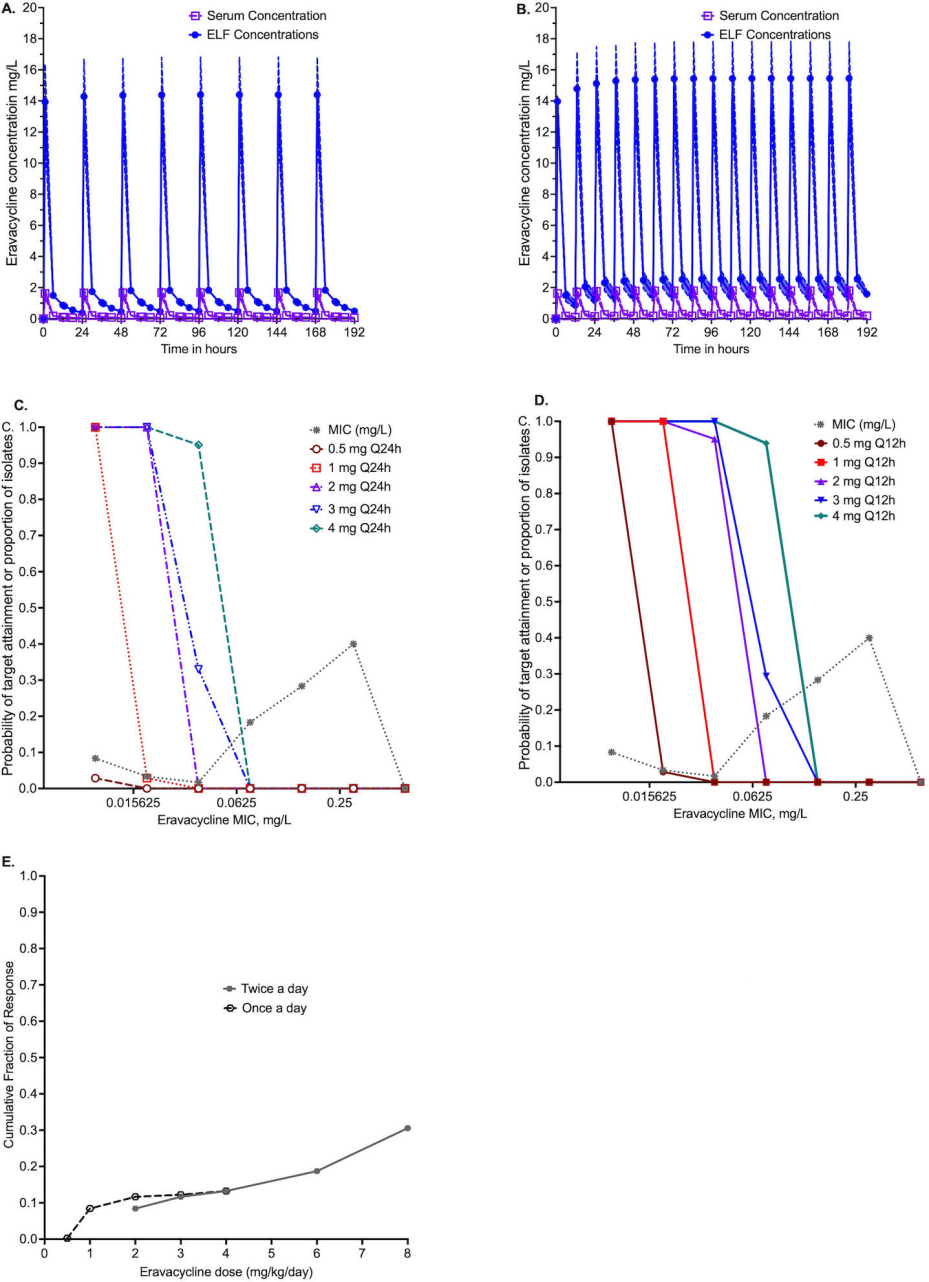
Dose-finding MCEs. (**A**) Concentration-time profiles in a 10,000 virtual patient simulation with eravacycline 1 mg/day dose in the serum and epithelial lining fluid (ELF). (**B**) Concentration-time profiles in a 10,000 virtual patient simulation with eravacycline 1 mg/day twice daily in the serum and ELF. (**C and D**) Probability of target attainment (AUC_0-24_/MIC of 3,588) in the lungs of patients with different eravacycline doses and once and twice-daily dosing schedules, considering the MIC distribution for 60 MAB isolates. (**E**) Cumulative fraction of response with eravacycline doses ranging from 0.5 to 4 mg/kg once or twice daily. Note, 4 mg/kg/day once daily will become a cumulative 8 mg/kg/day with a twice daily dosing schedule.

We examined the probability that eravacycline doses of 0.5, 1, 2, 3, and 4 mg/kg, once or twice a day, would achieve the EC_80_ AUC_0-24_/MIC of 3,588 in the lungs of patients with MAB pulmonary disease, in MCE of 10,000 virtual patients. The population PK estimates and variances generated by the MCE were similar to those entered into subroutine PRIOR of ADAPT, as shown in Table 4. The concentrations achieved with a dose of 1 mg/kg once versus twice a day are shown in Fig. 3A and B. The probability of target attainment (PTA) with different doses is shown in Fig. 3C. At the highest dose tested of 4 mg/kg twice a day (total dose of 8 mg/kg/day), the PTA falls below 90% at an MIC of 0.15 mg/L, versus the MIC_90_, which is one tube dilution higher. The cumulative fraction of response is shown in Fig. 3D, demonstrating that even at 8 mg/kg/day (administered as 4 mg/kg/day twice daily), the EC_80_ could be achieved in only 30.56% of patients.

**TABLE 4:** Comparison of the pharmacokinetic model parameters used in the simulations and the model output to show the accuracy of our approach

Parameter	Published by Ji et al. (18), used in the domain of input		MCE generated in 10,000 virtual subjects	
	Estimate	Inter-individual variability (%)	Estimate	Inter-individual variability (%)
Clearance in L/h	16.3	13.6	16.31	13.68
Central volume in L	3.88	116	3.797	111.9
Intercompartmental clearance (first compartment) in L/h	33.9	40	33.75	43.9
Peripheral volume one in L	39	55	39.1	54.43
Intercompartmental clearance (second compartment) in L/h	23.5	40	23.59	39.76
Peripheral volume two in L	122	40	122.3	40.49
ELF-to-plasma ratio	8.26			

## DISCUSSION

Tetracyclines, especially tigecycline, are an integral part of guideline-based therapy for MAB pulmonary disease (2, 10). In a previous PK/PD HFS-MAB study, tigecycline killed 1.23 log_10_ CFU/mL below *B_0_* (19). However, tigecycline is associated with considerable gastrointestinal adverse effects (5). Eravacycline, likely to have a lower adverse event profile, was tested here. While the drug had an effect, its efficacy (*E*_max_) was limited to 0.56 log_10_ CFU/mL below *B_0_*. This is considerably less than omadacycline, available in both oral and intravenous formulations, which demonstrated log_10_ CFU/mL below *B_0_* in the HFS-MAB, and has shown efficacy in the clinic with a better long-term safety profile (6, 8, 20–25). A second PK/PD finding is that, unlike other HFS-MAB studies, there was considerable sampling day-to-day variability in the EC_50_ and EC_80_, a concept which we have termed “wobbling” and is encountered in the HFS and in patients with slow-growing bacteria (6, 19, 26–37). The findings here suggest that the wobbling is not confined to slow-growing mycobacteria.

Here, the eravacycline MICs among the 60 MAB isolates, representing the three subspecies of MAB, were in the range of 0.0075 to 0.3 mg/L, and MIC_50_ and MIC_90_ were calculated as 0.15 and 0.3 mg/L, respectively. Previous studies report eravacycline MIC of the ATCC#19977 strain as 1 mg/L, which is >6-fold higher than we found in our repeated experiments (8). Similarly, the MIC_50_ and MIC_90_ have been reported as 1 and 4 mg/L, respectively, by Li et al. (9) and 0.5 and 1 mg/L, respectively, by Kaushik et al. (8). Another study by Brown-Elliot et al. (10) reported MIC_50_ as 0.12 mg/L and MIC_90_ as 0.5 mg/L, close to the values we report for the 60 isolates used in the current study. These MICs are similar to those of tigecycline and omadacycline (and in some instances better) when tested head-to-head, demonstrating that a good MIC pattern does not inform on whether a tetracycline has good efficacy or potency, which must be ascertained in PK/PD studies (5, 6, 8–10, 20–25).

In our static concentration experiments, eravacycline *E*_max_ (5.58 log_10_ CFU/mL) exceeded the standard-of-care drugs amikacin (2.65 log_10_ CFU/mL), clarithromycin (1.44 log_10_ CFU/mL), as well as omadacycline (3.22 log_10_ CFU/mL) (38). However, HFS-MAB studies, using dynamic concentrations, show a different order of microbial kill below *B_0_* of 0.56 log_10_ CFU/mL for eravacycline, 1.23 log_10_ CFU/mL for tigecycline, and 2.08 log_10_ CFU/mL for omadacycline (6, 19). Microbial kill below *B_0_* is a better comparison measure for ranking drugs compared with a raw *E*_max_ because of the following. *E*_max_ is calculated compared with the *E*_con_ of that specific sampling time point, where a drug can have a large *E*_max_ and still not kill below *B_0_*. Consider whereby the *E*_con_ is 9 log_10_ CFU/mL and the *E*_max_ is 4 log_10_ CFU/mL, but the *B_0_* was 4 log_10_ CFU/mL. In this example, while the *E*_max_ is high, the final bacterial burden is 1 log_10_ CFU/mL above *B_0_*. The discrepancy in terms of efficacy and potency between static concentration data versus HFS-MAB (which correlates with clinical findings) begs the question about the translational applicability of the static concentration data and MICs (6, 29).

The MCEs were performed with eravacycline doses for 0.5, 1, 2, 3, and 4 mg/kg, administered once or twice daily. The PTA of 4 mg/kg twice a day (8 mg/kg/day) fell below 90% at eravacycline of MIC 0.15 mg/L. The cumulative fraction of response at this highest dose was 30.56% of patients. This is despite that our MCE found AUCs that were a slight overestimate of those encountered in the serum of patients, and we used the 8.26-fold penetration factor into ELF. Moreover, our MIC distribution of our 60 isolates led to low MIC_50_ and MIC_90_ compared with the high MIC values reported by others (8, 9). This means that the cumulative fraction of response would fall even lower if the MICs published by others are used. Thus, eravacycline susceptibility testing methods need further optimization to remove these discrepancies between different laboratories and then update the MCEs in the future as more (and harmonized) data become available. Nevertheless, the optimal doses for MAB pulmonary disease are likely in the range that would not be tolerated by patients.

Among the limitations of the study is that we did not perform a dose-scheduling (dose fractionation) study to determine if the PTA could be improved with higher eravacycline doses, administered intermittently, which could potentially limit the side effects of the higher doses. While we assumed eravacycline efficacy is linked to AUC similar to other tetracyclines, a dose fractionation study would also have informed us on once versus twice daily administration, as there are some reports that a q12h dosing might be more effective (39, 40). Further, the eravacycline PK in the ELF may vary from serum based on differences in time to reach peak concentration and half-life due to a different clearance rate. However, with the emerging clinical evidence of oral omadacycline’s long-term efficacy and safety (6, 23–25), it is highly unlikely that eravacycline will be a drug of choice in the treatment of MAB pulmonary disease. Second, the use of one isolate (ATCC#19977) may not be generalizable to a larger patient population or calculation of a robust EC_80_ target. Third, the use of replicates may be important to account for biological variability in response. However, that adds considerable cost to HFS-MAB studies; resources that we did not have. Finally, we did not study AMR emergence to eravacycline in the HFS-MAB.

In summary, while eravacycline MICs and static concentrations suggest great potential and even activity better than omadacycline and tigecycline, the drug only killed 0.56 log_10_ CFU/mL below stasis in the HFS-MAB, which was the least among tetracyclines, such as omadacycline and tigecycline, that we have studied so far in the HFS-MAB. MICs and static concentrations should not be the sole adjudicator of drug’s (tetracyclines) efficacy against MAB and other NTMs.

## MATERIALS AND METHODS

### Bacteria, materials, and supplies

The standard laboratory strain of MAB (ATCC#19977) and 59 species-identified clinical isolates were used in the MIC experiments. Middlebrook 7H9 broth supplemented with 10% oleic acid-albumin-catalase–dextrose (OADC) (herein termed “7H9 broth”) was used to grow the bacteria, whereas cation-adjusted Mueller Hinton broth (herein termed “CA-MHB”) was used in the MIC experiments (41). The HFS-MAB experiment was performed using the ATCC strain, where 7H9 broth was used as the circulating medium, and Middlebrook 7H10 agar supplemented with 10% OADC (herein termed “7H10 agar”) was used for estimation of CFU. Eravacycline was purchased from the UT Health Science Center at Tyler campus pharmacy. The drug was reconstituted in 0.9% normal saline to get the desired concentrations to be used in the respective experiments. Hollow fiber cartridges (Cat#C8008) were purchased from FiberCell Systems (Frederick, MD, USA).

### Eravacycline MIC and static concentration-response studies

Eravacycline MICs of the ATCC laboratory strain and 59 clinical isolates were determined using the standard broth microdilution method (41). Inoculum was prepared by back diluting turbidity-adjusted (McF Standard 0.5 using a densitometer) logarithmic phase growth cultures to get a starting bacterial burden of ~10^5^ CFU/mL. Next, 198 µL of the inoculum was added to the 96-well microtiter plate prefilled with 2 µL (100×) eravacycline, with concentrations ranging from 0.0075 to 10 mg/L. Cultures were incubated at 30°C for 72 h, and visual MICs were recorded using an inverted mirror. The lowest eravacycline concentration inhibiting visible MAB growth was recorded as MIC. The experiment was performed twice, with two replicates for each concentration. The MICs were read by three individuals, and consensus between at least two readers was considered as the MIC value if a trailing effect was present.

For the static concentration-response studies, inoculum preparation and drug concentration range were the same as above. Two replicates per drug concentration were set up in a total volume of 5 mL, 50 µL (100×) of each concentration was added to the 4,950 µL of the inoculum. Next, after 72 h of incubation at 30°C under shaking conditions, 1 mL of the cultures was collected in a sterile centrifuge tube, washed twice with normal saline to remove the carry-over drug, and the pellets were resuspended in 1 mL saline and 10-fold serially diluted before inoculation on 7H10 agar for CFU estimation. Agar plates were sealed in a Ziplock bag to prevent drying, and CFUs were recorded after 72 h of incubation at 30°C.

### Eravacycline PK and PD

The HFS-MAB experiment used the standard laboratory strain, ATCC#19977. Twenty milliliters of the inoculum, prepared as described above, was loaded into the peripheral compartment of each of the ten HFS-MAB units. We used eight different human equivalent eravacycline doses to mimic the ELF AUC, one HFS-MAB unit per dose, and two nontreated control units. For clinical context, the peak (*C*_max_) plasma concentration following 1 mg/kg single intravenous infusion has been reported as 2.13 mg/L and the AUC_0-12_ as 4.31 mg*h*L^−1^ or a free-drug (*f*) AUC_0-12_ of 0.77 mg*h*L^−1^ (7). The eravacycline ELF-to-plasma concentration is proportional to 8.26 times free-drug concentration (18). Thus, 1 mg/kg will have an ELF AUC_0-24_ of 13.24 mg*h*L^−1^. Therefore, in this dose-response study, we used a range of low and high exposures (Table 2). The circulating medium in HFS-MAB was 7H9 broth plus 2% dextrose, and the dilution rate was set at 0.12 mL/min with an intended eravacycline half-life of 15 h. A previous study reported eravacycline’s half-life after a single ascending dose ranging between 12.7 and 25.6 h (42). The drug was infused over 1 h, once daily for 21 days, in the central compartment of the HFS-MAB using programmable syringe pumps. A recent study reported eravacycline solution stability at room temperature for up to 48 h when concentrations were measured using a reverse phase high-performance liquid chromatography (RP-HPLC) method (43). To capture the eravacycline concentration-time profile in the HFS-MAB units, systems were sampled on study day 1 before administration of the drug (pre-dose) followed by sampling at 1, 3, 9, 18, and 23.5 h post-dose. To estimate the bacterial kill in the eravacycline-treated systems and growth in the nontreated control, the central compartment of each HFS-MAB unit was sampled on days 0, 1, 2, 3, 4, 7, 10, 14, and 21. Samples were washed to remove the carry-over drug, 10-fold serially diluted, and cultured on 7H10 agar. CFUs were recorded after 72 h of incubation at 30°C.

### Drug concentration measurement

Eravacycline and azithromycin d3 (internal standard) were purchased from Toronto Research Chemicals (Toronto, Ontario, Canada). All other chromatographic or LC-MS/MS grade chemicals were purchased from Fisher Scientific. We employed a stable-isotope dilution liquid chromatography-electrospray ionization tandem mass spectrometry (LC-MS/MS) to determine eravacycline in the experimental samples (matrix 7H9 broth with 2% dextrose) from the HFS-MAB. LC-MS/MS analysis was performed using Waters Acquity UPLC connected to a Waters Xevo TQD mass spectrometer (Milford, MA). data were collected using MassLynx version 4.2 SCN985 software. Separation was achieved on a Waters Acquity UPLC BEH C18 1.7 µm 50 × 2.1 mm analytical column. All standard and internal standard (IS) stock solutions were prepared at 1 mg/mL in 100% methanol and stored at −20°C. Eravacycline was prepared with 50% MeOH. A six-point calibration curve, low-, mid-, and high-quality control samples (QCL QCM and QCH) were prepared by diluting the stock solution in 50% MeOH:water. In a 96-well plate, 20 µL of SC or QC was added to 180 µL of 7H9 + 2% dextrose broth, or 200 µL of sample were added to 20 µL of IS solution. An additional 400 µL of 20% MeOH was added, and the plate was mixed. The mobile phase A was a gradient mixture of aqueous FA/ammonium formate, and mobile phase B was methanol. The flow rate was 0.6 mL/min with a total run time of 3.5 min. Compounds were detected using ESI in MRM mode. The mass charge ratio for eravacycline was 559.2 > 89.3 and for azithromycin d3 was 752.5 > 158. The inter-day percent coefficient of variance (%CV) was 17.43, 11.33, 10.36, and 7.62 for the lower limit of quantitation (LLOQ), low-quality control (QCL), medium-quality control (QCM), and high-quality control (QCH), respectively. The inter-day % accuracy was 7.8, 0.3, −2.2, and −1.0, respectively. The intra-assay %CV ranged from 10.07 to 19.32 for the LLOQ, 5.93 to 13.16 for the QCL, 3.1 to 6.28 for the QCM, and 2.76 to 3.83 for the QCH. The intra-assay % accuracy ranged from −6.67 to 16.0 for the LLOQ, −5.72 to 10.94 for the QCL, −13.79 to 5.33 for the QCM, and −10.88 to 3.86 for the QCH. The lower limit of quantitation was 10 ng/mL.

### PK/PD analyses

Individual MICs were recorded in MS-EXCEL and used to calculate the MIC_50_ and MIC_90_ for 50% and 90% among the 59 clinical isolates. Measured drug concentrations were used to calculate the non-compartmental PK parameters, including the *C*_max_, AUC_0-24_, clearance (CL), the volume of distribution (Vd), and half-life (*t*_1/2_) in the HFS-MAB (Phoenix WinNonlin) (44). For the PD analysis, MAB log_10_ CFU/mL from each HFS-MAB unit on each sampling day versus AUC_0-24_/MIC was analyzed using the inhibitory sigmoid *E*_max_ model (26–28). From this equation, the concentration mediating 80% of *E*_max_ (EC_80_) was chosen as the target exposure in MCE-based *in silico* dose-finding experiments. GraphPad Prism version 10 was used for graphing the data.

### MCEs for *in silico* dose finding

The eravacycline population PK parameter estimates, and lung penetration indices entered into the subroutine PRIOR of ADAPT 5 model were those of Ji et al. (18). We examined doses (mg/kg) of 0.5, 1, 2, 3, and 4 mg/kg, once or twice a day, to achieve the EC_80_ AUC_0-24_/MIC of 3,588 in the lungs of patients with MAB pulmonary disease, in MCE of 10,000 virtual patients. Virtual subjects were dosed daily for over a week to achieve a steady state. The MCEs were performed assuming that the eravacycline PK increases linearly with the dose (42) in ADAPT 5, following steps described extensively before (6, 19, 26–29). The PTA and cumulative fraction of responses were calculated using the MIC distribution in our 60 MAB isolates.

## Data Availability

The raw data for the results presented in the article are available with the corresponding author, upon a reasonable request following UTHSCT’s data-sharing policy.
